# CCWeights: an R package and web application for automated evaluation and selection of weighting factors for accurate quantification using linear calibration curve

**DOI:** 10.1093/bioadv/vbab029

**Published:** 2021-10-28

**Authors:** Yonghui Dong, Tal Wachsman, Liat Morgan, Ehud Gazit, Rune Isak Dupont Birkler

**Affiliations:** Blavatnik Center for Drug Design, Tel Aviv University, Tel Aviv 69978, Israel

## Abstract

**Summary:**

The accuracy of any analytical method is highly dependent on the selection of an appropriate calibration model. Here, we present CCWeights, an R package for automated assessment and selection of weighting factors for accurate quantification using linear calibration curve. Additionally, CCWeights includes a web application that allows users to analyze their data using an interactive graphical user interface, without any programming requirements. The workflow and features of CCWeights are illustrated by the analyses of two datasets acquired by liquid chromatography-mass spectrometry (LC-MS). The resulting quantification table can be directly utilized for further model assessment and subsequent data analysis.

**Availability and implementation:**

CCWeights is publicly available on CRAN repository (https://cran.r-project.org/web/packages/CCWeights), with source code available on GitHub (https://github.com/YonghuiDong/CCWeights) under a GPL-3 license. The web application can be run locally from R console using a simple command “runGui()”. Alternatively, the web application can be freely accessed for direct online use at https://bcdd.shinyapps.io/CCWeights/.

**Supplementary information:**

Supplementary data are available at *Bioinformatics Advances* online.

## 1 Introduction

The accuracy of any analytical method is highly dependent on the selection of an appropriate calibration model. The most widely adopted model is unweighted linear regression (ULR), where the response (*y*-axis) is plotted against the corresponding concentration (*x*-axis) (da Silva *et al.*, 2015; Logue and Manandhar, 2018; Moosavi and Ghassabian, 2018). However, the wide concentration range used in modern bioanalytical assays (typically more than one order of magnitude) is susceptible to heteroscedasticity, where the variance increases with rising concentrations (Sonawane *et al.*, 2019). Larger variances associated with higher concentrations tend to influence (weight) the regression line more than that of the smaller variances present at lower concentrations. As a consequence, the accuracy of the analytical results, particularly at a lower concentration range, is impaired (Almeida *et al.*, 2002). For instance, it has been estimated that heteroscedasticity could lead to up to one order of magnitude precision loss in the low concentration region (Tellinghuisen, 2007).

A simple and effective way to account for heteroscedasticity and improve the accuracy over the selected concentration range is to use weighted linear regression (WLR) with an appropriate weighting factor (Almeida *et al.*, 2002; Sonawane *et al.*, 2019). Although WLR is a well-established statistics approach, the ‘Test-and-Fit’ strategy is still commonly used for the selection of calibration curves and weighting factors in the bioanalytical community due to its simplicity (Gu *et al.*, 2014; Moosavi and Ghassabian, 2018). Unfortunately, an improper weighting factor could be easily selected with the ‘Test-and-Fit’ strategy because it is based on the user’s subjective choice (Gu *et al.*, 2014). The correlation coefficient (*r*^2^) should not be used as the criteria in selecting weighting factor because the large variances present at high concentrations dominate the correlation coefficient calculation. In addition, it is suggested that a weighting factor should only be used for heteroscedastic data (Almeida *et al.*, 2002).

Although many commercial software, such as TraceFinder™ (Thermo Fisher, USA) and Targetlynx™ (Waters, USA), offer different weighting factors for users to choose for their gas chromatography or liquid chromatography mass spectrometry (GC- or LC-MS)-based targeted analyses, they do not provide functions to evaluate and select the appropriate weighting factor. To this end, we have developed CCWeights for automated selection of proper weighting factor for each individual compound, and analyte quantification using linear calibration curve. In particular, apart from the build-in weighting factors, CCWeights allows users to define their own weighting factors for model evaluation and analyte quantification. Furthermore, it provides a web interface which does not require any coding expertise.

## 2 Methods

**Fig. 1.**
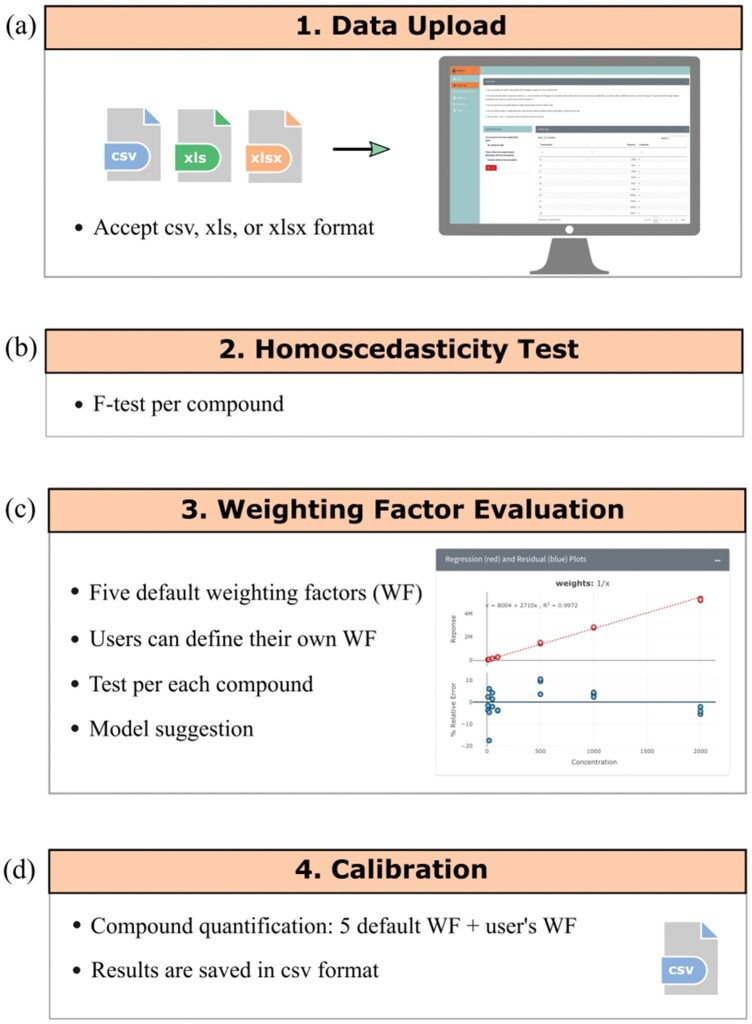
Schematic overview of CCWeights workflow. (a) Data can be uploaded in csv, xls or xlsx format in Data Upload panel. (b) Homoscedasticity test is then performed for each analyte. (c) Next, five default and one user-defined weighting factors (if available) are evaluated in order to select the optimum weighting factor for each analyte. (d) Finally weighted linear regression is performed for analyte quantification.

CCWeights is developed using R statistical language (R Core Team, 2020) and is released on both CRAN and GitHub. Additionally, a web application is built using R package Shiny (Beeley and Sukhdeve, 2018), allowing users to interactively analyze their data in a web browser without the need to download R or type any R commands. The analytical assay readout, exported as comma-separated values (.csv) or Microsoft Excel (.xls or .xlsx) format, is the starting point for the CCWeights pipeline (Fig. 1a). In order to help users familiarize themselves with the workflow and data format requirements, CCWeights is accompanied by two example datasets (Fig. 1a). The analyses of the two datasets are illustrated in Section 3. To make the web application more user-friendly, instructions are provided for each step.

As has been suggested that a weighting factor should only be used when homoscedasticity is not met for analytical data (Almeida *et al.*, 2002), CCWeights first tests data homoscedasticity by calculating the probability that the variance of measurements at the highest concentration level is smaller than the variance of measurements at the lowest concentration level using an *F*-test (Desharnais *et al.*, 2017a,b). The test of homoscedasticity is accepted when experimental *F*-value (*F*_exp_) is smaller than corresponding *F*-table value (*F*_tab_) at confidence of 99% (default value). Users can customize the confidence levels according to their studies (Fig. 1b). If the data are homoscedastic, weighting factor = 1 (1/*x*^0^, unweight linear regression) is suggested. Otherwise, five commonly used weighting factors, that is 1/*x*^0^, 1/*x*, 1/*x*^2^, 1/*y* and 1/*y*^2^, together with user-defined weighting factors (if present) are tested. By applying regression with different weighting factors on a set of calibration curve standard data, the best weighting factor could be identified by choosing the one generating the smallest sum of the absolute relative errors (SRE%) (Almeida *et al.*, 2002; Sonawane *et al.*, 2019). Interactive linear regression and residual plots are also provided for the user to evaluate different weighting factors. The figures can be downloaded in SVG format for further usage (Fig. 1c). Although CCWeights chooses the best weighting factor for the user, each targeted analyte is still quantified using all the tested weighting factors, which offers users the flexibility for further model evaluation. The resulting quantification table can be downloaded in csv format for subsequent data analysis (Fig. 1d).

## 3 Results

The features of CCWeights are illustrated by two examples: (i) targeted analysis without using any internal standards and (ii) targeted analysis with isotopically labeled counterparts as internal standards (Supplementary Files S1 and S2). Detailed sample preparation and data processing methods are provided in Supplementary File S3. Calibration results can be acquired by few clicks in the web application. In order to validate CCWeights results, the calibration curves were constructed using TraceFinder™ software (V5.1, Thermo) by manually selecting different weighting factors, that is 1/*x*^0^, 1/*x*, 1/*x*^2^, 1/*y*, 1/*y*^2^, and samples were quantified with each calibration model accordingly. The resulting linear regression models and quantification results were used to compare to those obtained from CCWeights, and CCWeights results are consistent with the ones obtained by TraceFinder™ (Supplementary File S4).

## 4 Conclusion

CCWeights is an efficient and easy-to-use R package and web application allowing automated optimized weighting factor selection for accurate quantification using linear calibration curve. It provides a user-friendly output which can be used for further model assessment and subsequent data analysis. It is important to note that although the workflow and features of CCWeights are illustrated using two LC-MS datasets, it can be used for any analytical data in practice.

## Funding

This study was supported by the Blavatnik Center for Drug Discovery, Tel Aviv University, funded by the Blavatnik Family Foundation.

*Conflict of Interest:* none declared.

## Supplementary Material

vbab029_Supplementary_Data
