# Supplementary material for: CCWeights: an R package and web application for automated evaluation and selection of weighting factors for accurate quantification using linear calibration curve
Source: Bioinform Adv. 2021 Oct 28;1(1):vbab029. doi: 10.1093/bioadv/vbab029 (PMC9710652; doi:10.1093/bioadv/vbab029)
Supplement: vbab029_Supplementary_Data [file vbab029_supplementary_data.zip › File S3. Materials and Methods for targeted LCMS analyses.docx]

**Material and Methods**

The first example dataset includes 6 metabolites, i.e., adenosine diphosphate (ADP), adenosine diphosphate ribose (ADPR), cyclic ADP-ribose (cADPR), malonyl CoA, nicotinamide adenine dinucleotide phosphate (oxidized form: NADP), nicotinamide adenine dinucleotide phosphate (reduced form: NADPH). The standard stock solution (10000 ng/mL) of each metabolite was prepared with fresh Milli-Q^®^ water. Then, mixed standard working solutions of 10, 20, 50, 100, 500, 1000, and 2000 ng/mL were used for calibration curve construction. The second example dataset contains 2 metabolites. i.e., adenosine monophosphate (AMP) and adenosine triphosphate (ATP). The same 7-level mixed calibration solutions were used for calibration curve construction except that 666 ng/ml of stable isotope labeled AMP (^15^N_5_-AMP) and ATP (^13^C_10_^15^N_1_-ATP) were spiked in each calibration solution, serving as internal standards. All the standards were purchased from Sigma-Aldrich.

LC-MS measurements were carried out by coupling of a Vanquish UHPLC to a Q-Exactive Orbitrap Focus (both Thermo Fisher Scientific). The separation was achieved using an Acquity UPLC HSS T3 Column, 100Å, 1.8 μm, 1 mm × 100 mm (Waters). UHPLC mobile phase A consisted of 5mM dibutylamine and mobile phase B was Acetonitrile (UHPLC grade, Sigma-Aldrich). The UHPLC gradient profile and flow rate are shown in Table 1. The column oven temperature was set at 25 °C, and the autosampler temperature was set at 10 °C. The injection volume was 5 μL and total run time was 15 min. The Q-Exactive ion source was equipped with a heated electrospray ionization (HESI) probe, and the Q-Exactive was tuned and calibrated using both positive and negative calibration solutions (Pierce^TM^ ESI Positive/Negative ion solutions, Thermo Scientific). The Q-Exactive was operated in targeted-selected ion monitoring (t-SIM) scan mode with a quadrupole isolation width of 3 *m/z* units. Metabolites were detected at negative ion mode with the mass resolution set to 35000. The automatic gain control (AGC) target was 1.0 × 10^6^ with the maximum injection time of 30 ms. The ionization source parameters were as follows: spray voltage, 2.7 kV; Aux gas heater temperature, 400°C; capillary temperature, 350°C; s-lens RF level, 40; Nitrogen was used as the sheath gas, auxiliary gas, and sweep gas at flow rates of 30, 13, and 0 a.u. (arbitrary units), respectively.

Data processing was performed using TraceFinder^TM^ software (version 5.1, Thermo Scientific). Peaks within a 5 ppm mass accuracy window of the target metabolite ions were integrated. The processed data were exported to Excel for CCWeights analysis. In order to validate CCWeights results, the calibration curves were constructed in parallel using TraceFinder^TM^ by manually selecting different weighting factors, i.e., 1/x^0^, 1/x, 1/x^2^, 1/y, 1/y^2^, and samples were quantified with each calibration model accordingly. The resulting linear regression models and quantification results were used to compare to those obtained from CCWeights.

Table 1. UHPLC gradient profile

| **Time** | **Solvent A** | **Solvent B** | **Flow rate (ml/min)** |
| --- | --- | --- | --- |
| 0.0 | 100% | 0.0% | 0.25 |
| 2.0 | 100% | 0.0% | 0.25 |
| 11.0 | 20% | 80% | 0.25 |
| 11.1 | 100% | 0.0% | 0.25 |
| 15.0 | 100% | 0.0% | 0.25 |
